# Prevalence of Gastric and Small-Intestinal Mucosal Injury in Elderly Patients Taking Enteric-Coated Aspirin by Magnetically Controlled Capsule Endoscopy

**DOI:** 10.1155/2019/1582590

**Published:** 2019-11-05

**Authors:** Feng Gao, Xue Chen, Jie Zhang

**Affiliations:** Digestive Department, Beijing An Zhen Hospital, Capital Medical University, Beijing 100029, China

## Abstract

**Objective:**

To investigate aspirin-related gastric and small-intestinal mucosal injury in elderly patients by magnetically controlled capsule endoscopy (MCCE).

**Methods:**

Patients taking enteric-coated aspirin attending the outpatient department of Beijing Anzhen Hospital, Capital Medical University, from September 2017 to July 2019 underwent MCCE to assess injury to the gastric and small-intestinal mucosa. The patients were divided into the elderly group (age ≥ 60 years) and middle-aged group (45 years ≤ age < 60 years), and their clinical data were evaluated.

**Results:**

Sixty-eight patients (34 per group) taking enteric-coated aspirin were recruited, and the elderly and middle-aged groups did not differ significantly in sex, history of smoking, history of alcohol consumption, body mass index, or accompanying diseases. In the elderly and middle-aged groups, the gastric Lanza scores were 2.0 (2.0, 3.0) and 2.0 (1.0, 3.0; *P* = 0.192), the numbers of patients with small-intestinal mucosal injuries (at least one erosion and/or ulcer) were 30 (88.2%) and 15 (44.1%; *P* < 0.001), the numbers of patients with more severe small-intestinal mucosal injuries (larger erosion and/or ulcer) were 11 (32.4%) and 3 (8.8%; *P* = 0.033), the numbers of patients with ileal erosion were 22 (64.7%) and 8 (23.5%; *P* = 0.001), and the durations of aspirin use were 30.0 (12.0, 120.0) and 10.5 (2.0–48.0) months (*P* = 0.007), respectively.

**Conclusions:**

The rate of small-intestinal mucosal injury was significantly higher in elderly than in middle-aged patients taking enteric-coated aspirin, especially the rate of ileal erosion. MCCE enables the monitoring of aspirin-related gastric and small-intestinal mucosal injury in elderly patients, which can guide treatment decision making.

## 1. Introduction

Aspirin inhibits platelet activation and thrombogenesis and is commonly used by patients with cardiovascular diseases [1, 2]. However, it also injures the gastrointestinal mucosa by exerting local and systemic effects, leading to erosion, ulceration, and bleeding [3, 4]. Furthermore, aspirin significantly increases the risks of gastric injury, ulceration, and serious bleeding, particularly in elderly individuals [5]. The introduction of capsule endoscopy (CE) and double-balloon enteroscopy has increased the rate of detection of aspirin-related small-intestinal mucosal injury [6–9]. The recent development of magnetically controlled capsule endoscopy (MCCE) combining examination of the upper gastrointestinal tract and small intestine can detect more diagnostic yield than esophagogastroduodenoscopy alone in patients with recurrent or refractory iron deficiency anemia [10], correctly predict safe discharge for patients with acute upper gastrointestinal bleeding [11], and also enable simultaneous screening for aspirin-related gastric and small-intestinal mucosal injuries [12]. Therefore, we investigated aspirin-related gastric and small-intestinal mucosal injury in elderly patients by MCCE.

## 2. Materials and Methods

### 2.1. Ethics

The study was approved by the local Ethics Board of Beijing Anzhen Hospital, Capital Medical University.

### 2.2. Subject Selection

Patients taking enteric-coated aspirin who were aged 45–75 years and attended Beijing Anzhen Hospital, Capital Medical University, from September 2017 to July 2019 were enrolled. The inclusion criterion was >1 month enteric-coated aspirin use. The exclusion criteria were dysphagia, known or suspected digestive tract obstruction, fistula, stenosis, or history of gastrointestinal surgery; presence of a cardiac pacemaker or metal implant; pregnancy or mental illness; positive C13 breath test result; severe heart, lung, liver, or renal dysfunction; and a history of using drugs affecting the study within 1 month before the examination, including (a) antibiotics, (b) probiotics, (c) drugs inhibiting the secretion of gastric acid, (d) cytokines, immunosuppressants, and cytotoxic agents, (e) hormones, and (f) other types of nonsteroidal anti-inflammatory drugs (NSAIDs).

### 2.3. MCCE

The MCCE system was produced by Shanghai Ankon Medical Technologies Co. Ltd. (Shanghai, China) and Ankon Technologies Co. Ltd. (Wuhan, China). The capsule contains a permanent magnet that can be guided manually by a magnet robot. The procedures for data recording and downloading are similar to those for other types of capsule.

### 2.4. Preparation for MCCE

The subjects ate a low-residue diet for 3 days before the examination and fasted from 7 p.m. on the night before the examination. Bowel preparation involved the consumption of four boxes of polyethylene glycol electrolyte powder (PGE; Staidson Beijing Biopharmaceutical Co., Ltd.) with 3.0 L clear water on the night before the examination and on the morning of the examination. Gastric preparation involved the consumption of simethicone power (5 g with 100 mL clear water 40 min before swallowing the capsule; Sichuan Jewelland Pharmaceutical Co., Ltd.), drinking of 300 mL clear water 30 min before swallowing the capsule, and drinking of 500 mL clear water immediately before swallowing the capsule [12].

### 2.5. MCCE Control Protocol

Each subject lay down on the console and swallowed the capsule. When the capsule entered the stomach, the operator controlled its position to visualize the stomach. After the capsule had entered the duodenum, the subject was permitted to leave the console and return home while wearing the check suit. The subjects returned the check suits, from which the data were exported to a computer workstation, and the images were analyzed by two experienced physicians using esNavi software [12].

### 2.6. Data Collection

Lanza scores for gastric mucosal injury are as follows: 0, no visible lesion; 1, mucosal erythema only; 2, 1–2 erosions; 3, 3–10 erosions; and 4, >10 erosions and/or ulcers [13]. Small-intestinal injury includes erosion and/or ulceration [14]. The controlled gastric examination time extended from the time at which the capsule entered the stomach to that at which it entered the duodenum. The small-intestinal transit time extended from the time at which the capsule entered the duodenum to that at which it entered the cecum [12].

### 2.7. Study Groups

The patients taking enteric-coated aspirin were divided into the elderly group (age ≥ 60 years) and the middle-aged group (45 years ≤ age < 60 years).

### 2.8. Statistical Analyses

Categorical data were compared using the chi-squared test or the Fisher exact test and presented as numbers (percentages). Normally distributed continuous data were compared using independent-sample *t*-tests and presented as means ± standard deviations (SDs). Nonnormally distributed continuous data were compared using the Mann-Whitney *U*-test and presented as medians with interquartile ranges (IQRs). *P* values < 0.05 were considered to indicate statistical significance. Statistical analysis was performed using SPSS software (ver. 22.0; IBM Corp., Armonk, NY).

## 3. Results

Sixty-eight patients (34 elderly, 34 middle aged) were enrolled in this study. The two groups showed no significant difference in sex; body mass index (BMI); gastric controlled examination time; small-intestinal transit time; or history of digestive ulcer, hypertension, diabetes mellitus, coronary artery disease, atrial fibrillation, cerebral infarction, or hyperlipemia (Table 1).

The results for the elderly and middle-aged groups were as follows: gastric Lanza score, 2.0 (2.0, 3.0) and 2.0 (1.0, 3.0; *P* = 0.192); gastric larger erosion and/or ulcer, 7 (20.5%) and 6 (17.6%) cases (*P* = 1.000); small-intestinal mucosal injury (at least one erosion and/or ulcer), 30 (88.2%) and 15 (44.1%) cases (*P* < 0.001); small-intestinal mucosal injury (larger erosion and/or ulcer), 11 (32.4%) and 3 (8.8%) cases (*P* = 0.033); duration of aspirin use, 30.0 (12.0, 120.0) and 10.5 (2.0–48.0) months (*P* = 0.007); positive result of fecal occult blood test, 8.8% and 5.9% (*P* = 1.000); haemoglobin level, 140.7 ± 12.2 g/L and 142.2 ± 10.7 g/L (*P* = 0.385); and urea level, 5.5 ± 1.4 mmol/L and 5.0 ± 1.4 mmol/L (*P* = 0.236), respectively. To further study the effect of the duration of aspirin on the small-intestinal mucosal injury, patients from the elderly group were divided into short period (≤12 months, 10 cases) and long period (>12 months, 24 cases), and the incidences of small-intestinal mucosal injuries (erosion and/or ulcer) were 50.0% and 79.2% (*P* = 0.089). In the middle-aged group, the incidences were 23.8% and 61.5% (*P* = 0.028).

The incidence of ileal erosion in the elderly group was significantly higher than that in the middle-aged group (64.7% versus 23.5%, *P* = 0.001). There was no difference in the incidences of duodenal erosion (22.5% versus 41.2%, *P* = 0.225), duodenal ulcer (2.9% versus 8.8%, *P* = 0.614), jejunal erosion (44.1% versus 29.4%, *P* = 0.314), jejunal ulcer (11.8% versus 2.9%, *P* = 0.356), and ileal ulcer (11.8% versus 5.9%, *P* = 0.673) between the two groups. The numbers of gastric Lanza scores between the elderly and middle-aged groups were as follows: score of 1—7 and 14; score of 2—15 and 10; score of 3—9 and 4; and score of 4—3 and 6, respectively (*P* = 0.286).

**Figure 1:**
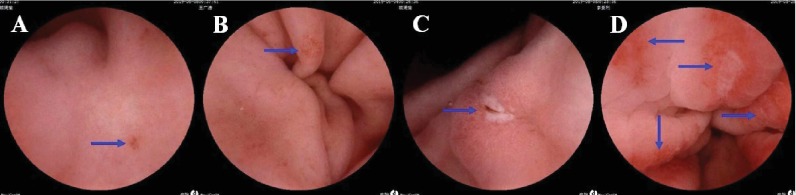
Magnetically controlled capsule endoscopic images of aspirin-related gastric mucosal injuries. Blue arrows, injuries. (a) Gastric antrum erosion. (b) Gastric antrum erosion. (c) Gastric antrum ulcer. (d) Gastric antrum ulcer and larger erosions.

**Figure 2:**
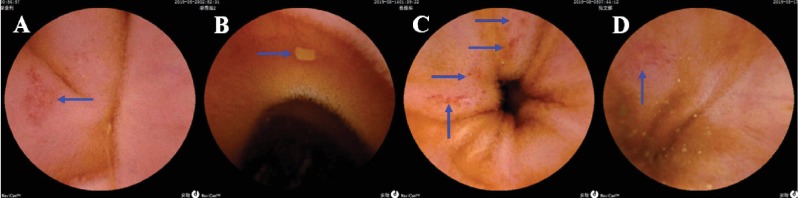
Magnetically controlled capsule endoscopic images of aspirin-related small-intestinal mucosal injuries. Blue arrows, injuries. (a) Jejunal erosion. (b) Jejunal ulcer. (c) Larger ileal erosions. (d) Ileal ulcer.

Magnetically controlled capsule endoscopic images of enteric-coated aspirin-related gastric and small-intestinal mucosal injuries are presented in Figures 1 and 2.

## 4. Discussion

Aspirin injures the mucosa of the digestive tract by inducing local and systemic actions that result in ulceration and bleeding. The incidence of gastroduodenal mucosal lesions is reportedly 48.4–63.1% versus 10.7–31.7% for gastroduodenal ulcers and 57.6% for small-intestinal mucosal lesions [3, 7]. The gastric mucosa of aging individuals and experimental animals show structural and functional abnormalities [5, 13, 14]. The gastric mucosa of elderly persons have impaired defenses, increased susceptibility to injury by a variety of noxious agents (*e*.*g*., aspirin, other NSAIDs, and ethanol), impaired angiogenesis, and delayed healing of erosions and ulcers. The mechanisms underlying these abnormalities include reduced mucosal blood flow causing hypoxia, deficiency of nerve growth factors in gastric endothelial cells, increased expression of phosphatase and tensin homolog, and reduced expression of vascular endothelial growth factor and survivin. MCCE is a new technique that enables examination of the stomach and small intestine simultaneously; it is noninvasive, comfortable, and safe; it does not require anesthesia; and it carries no risk of cross-infection [12, 15, 16]. Therefore, we investigated aspirin-related gastric and small-intestinal mucosal injuries in elderly Chinese patients by MCCE.

Among the elderly patients taking enteric-coated aspirin, 88.2% had at least one small-intestinal mucosal injury and 32.4% had larger erosions and/or ulcers. These rates were significantly higher than those of middle-aged patients, possibly due to longer durations of aspirin use by the elderly patients. The injury rates were not associated with patients' sex, BMI, histories of digestive ulcer, or accompanying diseases.

The mechanism of aspirin-related small-intestinal mucosal injury is unclear. Aspirin may reduce prostaglandin synthesis by inhibiting the activity of cyclooxygenase-1, which reduces mucous blood flow and synthesis and increases mucosal permeability, which may lead to injury of the small-intestinal mucosa. Aspirin may also destroy the integrity of intercellular junctions, increasing epithelial permeability, and injure the small-intestinal mucosa by damaging mitochondria. Aspirin may also accelerate gut mucosal invasion by bacteria by destroying the mucous layer, leading to the translocation of flora, inflammatory reaction, and injury to the small-intestinal mucosa [17–20]. In addition, older adults have altered gut microbiota compositions, which may play a key role in age-related inflammation and disease [21, 22].

The consumption of yogurt containing *Lactobacillus gasseri* twice daily for 6 weeks or *Lactobacillus casei* for 3 months was found to effectively treat aspirin-related small-bowel injury [23, 24]. In clinical practice, we have prescribed the probiotics (lactic acid bacteria capsules) for 2 months and found which probiotics can ameliorate aspirin-related small-intestinal mucosal injury in several patients, as determined by MCCE. And we are continuing a randomized controlled study of probiotics on these patients with small-intestinal mucosal injury.

The gastric Lanza score was slightly, but not significantly, higher in the elderly group than in the middle-aged group. This result may be due to the exclusion of patients taking other NSAIDs, antiplatelets, anticoagulants (*e.g.*, warfarin), or glucocorticoids, as well as those with *Helicobacter pylori* infection, from this study.

This study has several limitations. All of the subjects were from a single center, resulting in potential selection bias, and the sample was relatively small. We plan to increase the sample size and conduct multicenter prospective studies.

The rate of small-intestinal mucosal injury was significantly higher in elderly than in middle-aged patients taking enteric-coated aspirin, especially the rate of ileal erosion. MCCE enables the monitoring of aspirin-related gastric and small-intestinal mucosal injuries in elderly patients and can guide clinical decision making.

## Figures and Tables

**Table 1:** Demographic and clinical characteristics of patients taking enteric-coated aspirin.

Item	Elderly group	Middle-aged group	*P* value (chi-squared test, Fisher's exact test, independent bk?>samples *t*-test, or Mann-Whitney's *U*-test)
	*n* = 34	*n* = 34	
Age (mean ± SD, years)	65.5 ± 4.7	51.1 ± 5.4	*P* < 0.001
Male/female (*n*)	22/12	27/7	*P* = 0.280
BMI (mean ± SD, kg/m^2^)	25.5 ± 2.7	25.8 ± 3.2	*P* = 0.711
Aspirin using period (median (IQR), month)	30.0 (12.0, 120.0)	10.5 (2.0, 48.0)	*P* = 0.007
Gastric Lanza score (median (IQR))	2.0 (2.0, 3.0)	2.0 (1.0, 3.0)	*P* = 0.192
Gastric larger erosions and/or ulcer (*n* (%))	7 (20.5)	6 (17.6)	*P* = 1.000
Gastric controlled examination time (mean ± SD, min)	40.1 ± 7.4	42.4 ± 6.9	*P* = 0.208
Small-intestinal mucosal injury (at least one erosion and/or ulcer, *n* (%))	30 (88.2)	15 (44.1)	*P* < 0.001
Small-intestinal mucosal injury (larger erosions and/or ulcer, *n* (%))	11 (32.4)	3 (8.8)	*P* = 0.033
Small intestinal transit time (mean ± SD, min)	314.5 ± 122.2	281.2 ± 86.5	*P* = 0.199
Digestive ulcer history (*n* (%))	3 (8.8)	5 (14.7)	*P* = 0.709
Hypertension (*n* (%))	23 (67.6)	21 (61.8)	*P* = 0.800
Diabetes mellitus (*n* (%))	14 (41.2)	9 (26.5)	*P* = 0.305
Coronary artery disease (*n* (%))	17 (50.0)	12 (35.3)	*P* = 0.327
Atrial fibrillation (*n* (%))	1 (2.9)	1 (2.9)	*P* = 1.000
Cerebral infarction (*n* (%))	3 (8.8)	2 (5.9)	*P* = 1.000
Hyperlipemia (*n* (%))	11 (32.4)	13 (38.)	*P* = 0.800
Positive FOBT (*n* (%))	3 (8.8%)	2 (5.9%)	*P* = 1.000
Haemoglobin (mean ± SD, g/L)	140.7 ± 12.2	142.2 ± 10.7	*P* = 0.385
Urea (mean ± SD, mmol/L)	5.5 ± 1.4	5.0 ± 1.4	*P* = 0.236

SD, standard deviation; IQR, interquartile range; BMI, body mass index; FOBT: fecal occult blood test.

## Data Availability

Relevant raw data from this study are available upon request. Please contact the corresponding author.
